# Biomechanical research into lamina cribrosa in glaucoma

**DOI:** 10.1093/nsr/nwaa063

**Published:** 2020-04-11

**Authors:** Long Li, Fan Song

**Affiliations:** State Key Laboratory of Nonlinear Mechanics and Beijing Key Laboratory of Engineered Construction and Mechanobiology, Institute of Mechanics, Chinese Academy of Sciences, China; State Key Laboratory of Nonlinear Mechanics and Beijing Key Laboratory of Engineered Construction and Mechanobiology, Institute of Mechanics, Chinese Academy of Sciences, China; School of Engineering Science, University of Chinese Academy of Sciences, China

## Abstract

Glaucoma, a leading cause of irreversible blindness, poses a considerable public health challenge and burden. Mechanical models of the lamina cribrosa under elevated intraocular pressure at different scales, contributing significantly to uncovering the glaucomatous pathogenesis, are discussed. Meanwhile, the open issues and avenues for further development are highlighted.

## INTRODUCTION

**Figure 1.**
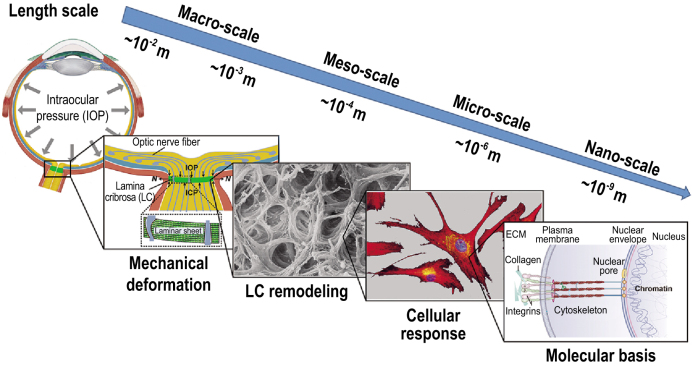
Mechanical responses of the LC to glaucoma at different length scales resulting from elevated IOP. The last three subfigures, from left to right, are adapted from [5], [6] and [7], respectively.

Glaucoma, from which ∼80 million people worldwide are now suffering, is the most common cause of irreversible blindness [1,2]. Its pathological mechanism is ascribed substantially to the irreversible deformation of the primary site—the lamina cribrosa (LC) of the sclera, induced by elevated intraocular pressure (IOP) rooting from the imbalance between production and drainage of aqueous humor [3]. It is this deformation that results in the damage to the optic nerve passing through the LC, as well as the capillaries, which then triggers the visual field defects, i.e. the occurrence of glaucoma. Controlling IOP to ensure that the deformation of the LC does not develop further is still the only proven treatment for glaucoma [4]. Obviously, the two mechanical factors, LC deformation and IOP change, play important roles in the progression of glaucoma. The other key factors in glaucoma are the multiscale structures and properties of the LC itself. Together they form a physical foundation for studying the development of glaucoma, thus giving rise to a common challenge for ophthalmology and biomechanics (Fig. 1). However, real-time observation and measurement of the state of the LC *in vivo*, as well as real-time monitoring of IOP, are scarcely possible at present for the clinical diagnosis and treatment of glaucoma. This means that building an appropriate mechanical model of the LC under elevated IOP remains the most efficient way to more fully understand the pathological mechanisms of glaucoma and to improve its clinical diagnosis and treatment.

## LAMINA CRIBROSA AS A BIOMECHANICAL STRUCTURE

As the primary site of glaucoma and the weakest part in the cornea-scleral envelope, the LC is, structurally, a sieve-like connective tissue composed of multilayered, elastic, anisotropic collagen fibrils [4,8,9]. This introduces strong orientation-dependent and highly nonlinear, viscoelastic attributes into the LC constitutive response and causes heterogeneous, rate- and time-dependent deformation fields [5]. Mechanically, the LC is a thin, clamped, circular plate with small deflections and is subjected to three loads: IOP and intracranial pressure (ICP), which act perpendicularly to the anterior and posterior surfaces of the LC, respectively; and in-plane pretension due to scleral expansion, which is parallel to the surface of the LC. Because ICP is constant under high IOP, the other two loads play the leading role in determining the state of the LC [10]. In response to these loads, the LC undergoes thickening/thinning, excavation, migration and scarring, accompanied by remodeling, synthesis and degradation of collagen fibrils.

## MODELING OF LAMINA CRIBROSA BIOMECHANICS

The first mechanical model of the LC was built using a thin film model [11]. Because the thin film model ignored the thickness and flexural resistance, it resulted in severe overestimation of deformation. Soon afterwards, many models were proposed, employing Kirchhoff's thin plate theory [12] and finite element method (FEM), to further study the deformation of the LC, but none of the results were in good agreement with relevant experiments [10]. This is because Kirchhoff's thin plate theory inherently ignores the shear effects in the LC, while FEM can hardly obtain the accurate deformation of the LC as the optic nerve channels and the laminar sheets in the LC both have very small dimensions and tangled structures.

Recently, a new mechanical model of the LC was developed based on Reissner's thin plate theory [10]. Because the model abandons Kirchhoff's hypothesis of straight normals, it provides all the stresses and deformations present in the LC under elevated IOP, which have proven to be in good agreement with the existing experiments. This model first presents the shear effects on the deformation of the optic nerve channels and laminar sheets in the LC. As a result of the IOP-induced complex deformation, for the pores close to the LC center, their shapes on the anterior and posterior surfaces remain circular but with increased and decreased areas, respectively. These LC pores ultimately change into straight horns from initially straight cylinders, because the deformation caused by the shear strain is negligible. However, the deformation of pores close to the LC rim differs from that of pores close to the LC center. In detail, the shapes of the pores close to the LC rim change from circular to oval, the areas of the pores on the anterior and posterior surfaces increase and decrease, respectively, and the channels deform into tortuous horns from initially straight cylinders (subfigure 2 of Fig. 1, from left to right), which was confirmed in recent experiments [13]. Meanwhile, the deformation of the pores close to the LC rim was demonstrated to be much greater than that close to the LC center. The IOP-induced compression of laminar sheets and distortion of laminar pores are substantial enough to impede the axoplasmic flow in the retinal ganglion cell axons and the blood supply in the capillaries, leading to ischemia-hypoxia damage, growth factor deprivation, and altered cellular and molecular responses, thus contributing to the symptoms of glaucoma [14]. The ratio of optic cup to disc diameters (C/D), an empirical parameter, is the most useful quantitative index frequently used to detect glaucoma and assess its progression [1,8]. This model demonstrates that the critical C/D, above which glaucoma should be suspected, is the point that the largest deformation gradient in the LC reaches. It also presents a possible pathological mechanism for glaucoma that occurs despite normal IOP and explains why glaucomatous visual loss starts in the periphery and progressively advances to involve the central vision.

The connective tissues and cells in the LC also experience and respond to the IOP-induced deformation. A few models have proposed that a preferred radial orientation of the collagen fibrils in the periphery of the LC can reinforce the LC against high transverse shear forces and reduce the bending deformations of the LC. In adapting to the residual strains between collagen fibrils, IOP elevation can lead to LC thickening and stiffening, because of an increase in collagen fibril mass and the recruitment of adjacent neural canal tissue into the LC [5]. This means that the LC undergoes self-adaptation to the glaucomatous mechanical environments through a biomechanical feedback mechanism. In particular, the transmission of elevated IOP to the laminar microstructures of the LC is indicated to result in tremendous amplification of the stresses and strains that the cells in the lamina are experiencing [4], which should be associated with the stress concentration near the pores in the LC.

## FURTHER PERSPECTIVES

Despite the fact that considerable progress in biomechanical research has contributed to the understanding of the pathological mechanisms of glaucoma and the improvement of its clinical diagnosis and treatment, the biomechanical studies of glaucoma still face multiple challenges. Further models should precisely reflect the damage evolution of optic nerve and capillaries under elevated IOP, and relate the damage to the visual field defects, wherein the effects of the stress concentration near the channels and pores on the optic nerve and capillaries passing through them cannot be overlooked, and fluid-structure interactions, as well as viscous and anisotropic material properties also need to be taken into account. Because glaucoma is usually asymptomatic until severe visual problems arise, the relationship between the deformation and the damage of the optic nerve and capillaries, near to the critical point of the C/D, needs to be identified to develop and improve methods for the early diagnosis of glaucoma. In particular, building multiscale biomechanical models of glaucoma is very beneficial for better understanding the interdependent behaviors and responses from the molecules to the cells to the tissues and the correlations between glaucomatous insults at different length scales. For example, the molecular and cellular responses to glaucomatous insults are collectively associated with identifying potential therapeutic targets in clinical practice. Additionally, it is important to note that glaucoma is a multifactorial disease. Accumulating evidence has revealed that, in addition to elevated IOP, there are many other pathophysiological factors including vascular dysregulation, hypoxia, excitotoxicity, oxidative stress, mitochondrial dysfunction, and autoimmunity involved in the progression of retinal ganglion cell death and glaucomatous optic neuropathy. It is the complex interplay of these multiple factors that poses a major challenge in the prevention and treatment of glaucoma.
